# The Diagnostic Value of Susceptibility-Weighted Imaging in Preoperative Grading of Glial Tumors

**DOI:** 10.7759/cureus.89757

**Published:** 2025-08-10

**Authors:** Ozgur Genc, Omer N Tabakci, Ender Uysal, Saime A Sahin, Canan T Tanık

**Affiliations:** 1 Department of Radiology, IAU VM Medical Park Florya Hospital, Istanbul, TUR; 2 Department of Radiology, Kocaeli State Hospital, Istanbul, TUR; 3 Radiology, University of Health Sciences Turkey, Antalya Training and Research Hospital, Antalya, TUR; 4 Department of Neurosurgery, Sisli Hamidiye Etfal Research and Training Hospital, Istanbul, TUR; 5 Department of Pathology, Sisli Hamidiye Etfal Research and Training Hospital, Istanbul, TUR

**Keywords:** advanced mri techniques, grading of glioma, high-grade glioma, intratumoral susceptibility signals, itss quantification, low-grade glioma, mri-based tumor analysis, noninvasive glioma grading, preoperative brain mri, susceptibility-weighted imaging (swi)

## Abstract

Introduction

Susceptibility-weighted imaging (SWI) is a noninvasive MRI technique that detects microvascular features such as hemorrhage and neovascularization in brain tumors. Intratumoral susceptibility signals (ITSS) observed on SWI have been associated with tumor grade, particularly in gliomas. This study aimed to investigate the correlation between SWI-derived ITSS and tumor histological grades, and to evaluate the diagnostic performance of SWI in glioma grading.

Materials and methods

This retrospective single-center observational study included 44 adult patients (22 males, 22 females; mean age: 41.2 ± 18.5 years) with histologically confirmed glial tumors who underwent preoperative MRI with SWI between 2015 and 2020. ITSS were visually graded on SWI sequences as follows: Grade 0 (no ITSS), Grade 1 (1-5 foci), Grade 2 (6-10 foci), and Grade 3 (>10 foci). Grading was performed for both the entire tumor and the tumor center. Tumor grades were classified according to the 2021 WHO criteria as low-grade (Grades I-II) or high-grade (Grades III-IV). Statistical analysis included Mann-Whitney U, Kruskal-Wallis, and Spearman’s correlation tests.

Results

ITSS was present in 34 patients (77.3%). Among ITSS-positive cases, 23 of 34 patients (67.6%) had high-grade gliomas, while 11 patients (32.3%) had low-grade gliomas. All ITSS-negative patients had low-grade gliomas. SWI grades showed a significant positive correlation with WHO tumor grades (r = 0.479, p = 0.001 for whole tumor; r = 0.461, p = 0.002 for tumor center). SWI grading differed significantly across WHO grades (p = 0.001). In whole-tumor evaluation, SWI achieved 74% sensitivity and 71% specificity for predicting high-grade gliomas. Tumor center evaluation yielded 65% sensitivity, 76% specificity, 75% positive predictive value (PPV), and 66% negative predictive value (NPV). ITSS-positive patients were significantly older than ITSS-negative patients (p = 0.001).

Conclusions

SWI-detected ITSS strongly correlates with glioma histopathological grade and patient age. Given its diagnostic performance, reproducibility, and contrast-free nature, SWI represents a valuable noninvasive tool for glioma grading and may be integrated into routine preoperative MRI protocols.

## Introduction

Glial tumors represent one of the most prevalent primary neoplasms of the central nervous system, accounting for approximately one-third of all intracranial tumors. The World Health Organization (WHO) classifies gliomas into four grades (I-IV) based on histopathological features, including cellular atypia, mitotic activity, microvascular proliferation, and necrosis. This grading system is critical for guiding treatment strategies and estimating prognosis. However, it requires tissue sampling via biopsy or resection procedures that may be contraindicated or infeasible in certain clinical settings [1-3].

MRI remains the cornerstone of brain tumor evaluation, given its superior soft-tissue contrast and anatomical resolution. Nevertheless, conventional sequences, such as contrast-enhanced T1-weighted or T2-weighted imaging, are limited in their ability to reflect tumor microvascularity or biological behavior accurately. To overcome these limitations, advanced MRI techniques such as susceptibility-weighted imaging (SWI) have been increasingly applied in neuro-oncology [4,5]. SWI is a high-resolution, gradient-echo MRI technique that combines magnitude and phase data to enhance susceptibility effects from paramagnetic substances such as deoxyhemoglobin, ferritin, and hemosiderin. Compared to conventional T2-weighted imaging, SWI provides improved visualization of venous anatomy, microhemorrhages, and vascular proliferation. One of the most relevant features observed on SWI in gliomas is the intratumoral susceptibility signal (ITSS), which manifests as linear or punctate hypointense foci [6-8].

ITSS is believed to reflect intratumoral microvascular proliferation and microscopic hemorrhage, each of which is a hallmark feature of tumor aggressiveness. Several studies have shown that ITSS is more frequently observed in high-grade gliomas, whereas it is typically absent or minimal in low-grade lesions. For instance, Park et al. have reported a significant correlation between ITSS burden and glioma histological grade. Similarly, Qin et al. have demonstrated that semi-quantitative SWI grading aligned closely with tumor vascular density [9-11].

Given that preoperative tumor grading influences surgical planning, radiotherapy decisions, and patient counseling, imaging modalities capable of estimating tumor grade noninvasively are of substantial clinical value, particularly when biopsy is delayed or contraindicated. In this context, SWI offers a promising tool for early glioma stratification and clinical decision support [5,9,12]. This study aimed to assess the diagnostic performance of SWI-derived ITSS in predicting glioma grade based on the 2021 WHO classification [1].

## Materials and methods

This retrospective observational study included 44 adult patients who were evaluated between 2015 and 2020 at the radiology and neurosurgery departments of a tertiary university hospital. Ethical approval was obtained from the institutional review board, and the study was conducted in accordance with the Declaration of Helsinki.

The inclusion criteria were as follows: histopathologically confirmed diagnosis of a glial tumor, availability of a preoperative MRI study with an SWI sequence of sufficient quality, and complete access to clinical data, including age and sex. Exclusion criteria included poor image quality, lack of SWI sequence, absence of histopathological confirmation, secondary malignancies, and diagnoses made without tissue sampling (e.g., radiological or clinical only). In addition, non-glial intra-axial lesions such as central nervous system lymphomas, brain metastases, or infectious/inflammatory masses were excluded to ensure pathological homogeneity and to prevent potential bias in SWI signal interpretation.

Imaging protocol

All MRI examinations were performed using a 1.5 Tesla Siemens Avanto system (Siemens Healthineers, Erlangen, Germany). The imaging protocol included axial T1-weighted, T2-weighted, fluid-attenuated inversion recovery (FLAIR), post-contrast T1-weighted, and SWI sequences. The acquisition parameters for the SWI sequence were as follows: TE = 40 ms, TR = 50 ms, flip angle = 15°, field of view = 240 mm, matrix size = 256 × 256, slice thickness = 2 mm, and 20 slices in total. All imaging data were evaluated in DICOM format via the institutional PACS (Picture Archiving and Communication System). Artifacts were excluded from analysis. Brainstem and cerebellar tumors were not included in the study.

Radiological evaluation

All SWI sequences were independently reviewed by a neuroradiologist with over 10 years of experience, who was blinded to the clinical and histopathological data. Evaluations focused on the slice showing the highest density of ITSS. ITSS was defined as hypointense, linear, or dot-like foci within the tumor.

A visual scoring system was applied as follows: Grade 0: No ITSS; Grade 1: 1-5 ITSS foci; Grade 2: 6-10 ITSS foci; and Grade 3: >10 ITSS foci

Grading was conducted separately for the entire tumor and the tumor center. For central evaluation, a circular region of interest (ROI) was placed at the geometric center of the tumor, with a radius equal to half the maximum tumor diameter. Only ITSS within this ROI were considered. This dual-assessment approach was adapted from previously published protocols.

Peritumoral edema, cystic degeneration, and necrosis were documented but excluded from statistical analysis, as the primary objective was to evaluate the correlation between ITSS and histological tumor grade. 

Histopathological evaluation

Histopathological diagnoses were performed by experienced neuropathologists using tissue samples obtained through surgical resection or stereotactic biopsy. Tumors were classified according to the 2021 WHO classification system as low-grade (Grade I-II) or high-grade (Grade III-IV) [1]. While histological subtypes (e.g., diffuse astrocytoma, glioblastoma, oligodendroglioma) were noted, the analysis focused solely on tumor grade. Molecular data such as IDH mutation status and MGMT promoter methylation were excluded due to limited availability. In cases where multiple specimens were obtained from a single patient, the highest histological grade was used for statistical analysis.

Statistical analysis

All statistical analyses were conducted using SPSS Statistics version 24.0 (IBM Corp., Armonk, NY). Descriptive statistics included means, standard deviations (SDs), medians, and interquartile ranges (IQRs). The Spearman rank correlation coefficient was used to assess associations between SWI grades and histological tumor grades. Mann-Whitney U and Kruskal-Wallis tests were used for group comparisons. A p-value less than 0.05 was considered statistically significant. Median SWI grades for both the tumor center and the whole tumor were calculated separately for ITSS-positive and ITSS-negative patient groups, and group-wise comparisons were performed using the Mann-Whitney U test.

## Results

A total of 44 patients were included in the study, with an equal gender distribution: 22 females (50%) and 22 males (50%). The mean age of the cohort was 41.18 ± 18.53 years (range: 5-78 years). When stratified by sex, the mean age was 36.90 ± 16.27 years for males and 45.45 ± 20.01 years for females. However, the difference in mean age between sexes was not statistically significant (p>0.05). According to the 2021 WHO classification of central nervous system tumors, 16 patients (36.4%) were diagnosed with Grade IV gliomas, 14 (31.8%) with Grade II gliomas, and seven (15.9%) each with Grade I and Grade III gliomas (Table 1).

**Table 1:** Histopathological subtypes of glial tumors in the study cohort WHO: World Health Organization

WHO grade	Histopathological subtype	Number of cases (n)
Low-grade gliomas (Grade I–II)	Diffuse astrocytoma	4
	Pilocytic astrocytoma	3
	Oligodendroglioma	8
	Ganglioglioma	2
	Pleomorphic xanthoastrocytoma	2
	Subependymal giant cell astrocytoma (SEGA)	2
	Subtotal	21
High-grade gliomas (Grade III–IV)	Anaplastic astrocytoma	4
	Anaplastic oligodendroglioma	3
	Glioblastoma (GBM)	16
	Subtotal	23
	Total	44

Although four SWI grades (0 to 3) were predefined in the evaluation system, no patient in the study population was assigned to SWI Grade 2 (6-10 blooming foci). Thus, only SWI Grades 0, 1, and 3 were observed in this cohort. 

ITSS, defined as SWI Grade ≥1, was observed in 34 patients (77.3%). Among ITSS-positive patients, 23 (67.6%) had high-grade gliomas (Grades III-IV), while 11 (32.3%) had low-grade gliomas (Grades I-II). Conversely, all of the ITSS negative patients (SWI Grade 0) had low-grade gliomas (Table 2).

**Table 2:** Distribution of histopathological WHO glioma grades according to SWI grades SWI Grade 0: Absence of intratumoral susceptibility signals (ITSS); no visible blooming artifacts on SWI. SWI Grade 1: Minimal ITSS (1-5 dot-like or linear blooming foci). SWI Grade 2: Moderate ITSS (6-10 blooming foci). SWI Grade 3: Marked ITSS (>10 blooming foci). WHO grades were determined according to the 2021 WHO classification of CNS tumors (Louis et al.) CNS: central nervous system; ITSS: intratumoral susceptibility signals; SWI: susceptibility-weighted imaging; WHO: World Health Organization

SWI grade	Definition	Grade I	Grade II	Grade III	Grade IV	Total
0	No ITSS – no blooming artifacts	3	7	0	0	10
1	1-5 dot/linear blooming foci (minimal)	4	7	2	1	14
2	6-10 dot/linear blooming foci (moderate)	0	0	0	0	0
3	>10 dot/linear blooming foci (dense)	0	0	5	15	20
Total		7	14	7	16	44

The mean age of ITSS-positive patients was significantly higher than that of ITSS-negative patients (49.47 ± 15.49 vs. 32.09 ± 17.56 years; p = 0.001). Similarly, patients with high-grade gliomas had a significantly higher mean age compared to those with low-grade tumors (47.17 ± 18.14 vs. 34.61 ± 16.70 years; p = 0.023).

There was a statistically significant positive correlation between SWI grades and WHO tumor grades. In the whole-tumor evaluation, Spearman’s rank correlation coefficient was r = 0.479 (p = 0.001), and in the tumor center evaluation, it was as follows: r = 0.461 (p = 0.002). These findings indicate that higher SWI grades are strongly associated with higher histopathological tumor grades. The Kruskal-Wallis test also revealed significant differences in SWI grades across the four WHO tumor grades for both whole-tumor and tumor center assessments (Table 3), further supporting the association between SWI findings and tumor aggressiveness.

**Table 3:** Kruskal-Wallis test results comparing SWI grades across WHO tumor grades Statistically significant differences were observed in both tumor center and whole tumor evaluations SWI: susceptibility-weighted imaging; WHO: World Health Organization

Comparison	H statistic	P-value
SWI grade (tumor center) vs. WHO grade	H = 17.127	0.001
SWI grade (whole tumor) vs. WHO grade	H = 19.035	0.001

Patients with ITSS positivity (n = 34) were significantly older and had higher SWI grades compared to ITSS-negative patients (n = 10) (p = 0.001 for all comparisons). Median SWI grades were higher in both the tumor center and the whole tumor in the ITSS-positive group (Table 4).

**Table 4:** Mann–Whitney U test results comparing age and SWI grades between ITSS-positive and negative patients Median SWI grades were calculated separately for the tumor center and the whole tumor, allowing for side-by-side comparison of susceptibility patterns between groups ITSS: intratumoral susceptibility signals; SWI: susceptibility-weighted imaging; SD: standard deviation

Variable	ITSS-positive (n = 34)	ITSS-negative (n = 10)	U statistic	P-value
Age, years, mean ± SD	56.3 ± 9.1	43.6 ± 10.4	114.5	0.001
SWI grade - tumor center, median)	2	1	138.5	0.001
SWI grade – whole tumor, median	2	1	122	0.001

To evaluate the diagnostic accuracy of SWI in distinguishing high-grade from low-grade gliomas, performance metrics were calculated. In the whole-tumor assessment, SWI demonstrated a sensitivity of 74%, specificity of 71%, positive predictive value (PPV) of 74%, and negative predictive value (NPV) of 71%. In the tumor center assessment, sensitivity was 65%, specificity 76%, PPV 75%, and NPV 66%. These findings endorse the potential clinical utility of SWI as a noninvasive imaging marker in glioma grading.

## Discussion

This study evaluated the diagnostic utility of SWI-derived ITSS in predicting glioma grade, based on the 2021 WHO classification [1]. The findings confirm that higher SWI grades are significantly associated with high-grade gliomas. This is consistent with previous studies indicating that ITSS reflects key pathological features of tumor aggressiveness, including microvascular proliferation, microhemorrhages, and calcifications, each recognized as a hallmark of high-grade gliomas [9-11].

A strong positive correlation was observed between SWI grades and WHO tumor grades (r = 0.479, p = 0.001 for the whole tumor; r = 0.461, p = 0.002 for the tumor center), with SWI Grade 3 being particularly predictive of high-grade gliomas. These results corroborate earlier findings suggesting that SWI serves as a valuable noninvasive tool for preoperative tumor characterization [13,14]. In this cohort, 67.6% of ITSS-positive patients had high-grade gliomas, whereas all of the ITSS-negative patients had low-grade tumors. Thus, the presence and degree of ITSS can serve as a reliable imaging surrogate for tumor biological behavior [15-17]. Age-stratified analysis further revealed that both ITSS positivity and high-grade gliomas were significantly more frequent in older patients. This may reflect age-related vascular fragility and neovascularization, which are known to enhance susceptibility effects on SWI [18].

The diagnostic performance of SWI in distinguishing low- from high-grade gliomas-74% sensitivity and 71% specificity in the whole-tumor assessment, is comparable to previously published results from SWI-based and radiomics-supported analyses [19-21]. In addition, advancements in radiomics and artificial intelligence (AI) have opened new avenues for SWI interpretation. Deep learning models have shown superiority over conventional visual scoring systems by reducing interobserver variability and enabling automated, reproducible quantification of ITSS [22,23]. For instance, Liu et al. demonstrated that AI models trained on balanced datasets achieved high accuracy in glioma grading, even in low-sample-size scenarios [21]. Similarly, SWI-derived radiomic features have proven valuable in differentiating glioma subtypes and predicting IDH mutation status when combined with deep learning algorithms [24].

Despite these promising results, the study has certain limitations. The sample size was relatively small (n = 44), and the retrospective, single-center design may limit generalizability. Another limitation is that our cohort lacks tumors classified as SWI Grade 2. Additionally, molecular diagnostic parameters-such as IDH mutation, MGMT promoter methylation, and 1p/19q codeletion-were unavailable for most patients, precluding integration with molecular glioma classification frameworks. Future research should aim to validate these findings in larger, multicenter cohorts and explore combined imaging-genomic models for more precise glioma stratification.

To sum up, SWI grading based on the presence and intensity of ITSS is significantly correlated with histological glioma grade and patient age. SWI is a promising, noninvasive imaging biomarker with reproducible diagnostic performance and strong potential for integration into standard preoperative MRI protocols. With the aid of AI and radiomics, SWI may emerge as a cornerstone modality in future glioma diagnosis, grading, and prognostication.

## Conclusions

SWI offers a noninvasive, contrast-free modality for evaluating ITSS, which are significantly correlated with glioma histological grade and patient age. This study demonstrated that higher SWI grades are strongly associated with high-grade gliomas, endorsing the utility of ITSS as a reliable surrogate marker of tumor aggressiveness. Given its diagnostic accuracy, reproducibility, and compatibility with routine preoperative MRI workflows, SWI represents a valuable adjunct in the imaging-based grading of gliomas. Future prospective studies with larger sample sizes and integrated molecular profiling are warranted to validate these findings and further elucidate the role of SWI in the comprehensive assessment of glioma biology.
